# Identification of key protein-coding genes and lncRNAs in spontaneous neutrophil apoptosis

**DOI:** 10.1038/s41598-019-51597-9

**Published:** 2019-10-22

**Authors:** Nan Jiang, Xinzhuo Zhang, Yancheng He, Bo Luo, Chengcheng He, Yu Liang, Jingyuan Zeng, Wei Li, Yujun Xian, Xiaoli Zheng

**Affiliations:** 1 School of Basic Medicine, Southwest Medical University, No. 1 Xianglin Road, Luzhou, Sichuan China; 2 School of International Education, Southwest Medical University, No. 1 Xianglin Road, Luzhou, Sichuan China; 3 People’s Hospital of Zhongjiang, Deyang, Sichuan China; 4 The Affiliated Hospital (TCM) of Southwest Medical University, Luzhou, China

**Keywords:** Apoptosis, Apoptosis, Immunogenetics, Immunogenetics

## Abstract

Polymorphonuclear leukocytes (PMNs) are the most abundant cells of the innate immune system in humans, and spontaneous PMN apoptosis plays crucial roles in maintaining neutrophil homeostasis and resolving inflammation. However, the detailed mechanisms of spontaneous PMN apoptosis remain to be elucidated. By analysis of the public microarray dataset GSE37416, we identified a total of 3050 mRNAs and 220 long non-coding RNAs (lncRNAs) specifically expressed during PMN apoptosis in a time-dependent manner. By short time-series expression miner (STEM) analysis, Gene Ontology analysis, and lncRNA-mRNA co-expression network analyses, we identified some key molecules specifically related to PMN apoptosis. STEM analysis identified 12 gene profiles with statistically significance, including 2 associated with apoptosis. Protein-protein interaction (PPI) network analysis of the genes from 2 profiles and lncRNA-mRNA co-expression network analysis identified a 12-gene hub (including NFκB1 and BIRC3) associated with apoptosis, as well as 2 highly correlated lncRNAs (THAP9-AS1, and AL021707.6). We experimentally examined the expression profiles of two mRNA (NFκB1 and BIRC3) and two lncRNAs (THAP9-AS1 andAL021707.6) by quantitative real-time polymerase chain reaction to confirm their time-dependent expressions. These data altogether demonstrated that these genes are involved in the regulation of spontaneous neutrophil apoptosis and the corresponding gene products could also serve as potential key regulatory molecules for PMN apoptosis and/or therapeutic targets for over-reactive inflammatory response caused by the abnormality in PMN apoptosis.

## Introduction

Human polymorphonuclear leukocytes (PMNs or neutrophils) are the most abundant white cells in the blood and serve as the first line of host defense. They are terminally differentiated cells with an extremely short lifespan (8–20 hours in circulation and 1–4 days in tissue) and a daily turnover of 10^11^ cells^1^. Spontaneous apoptosis of neutrophil plays an important regulatory role in this dramatic turnover^2^. It is generally agreed that the death of PMN under physiological condition is spontaneous apoptosis, also known as constitutive apoptosis^3^, which can be mimicked *in vitro* by culturing the cell in the absence of sufficient amount of survival cytokines^4^. It was believed that mature PMNs were nearly transcriptionally inert and only a few proteins were selectively expressed^5^. However, a systems biology approach revealed that the lifespan of PMN could be regulated at the transcriptional level^6^. Multiple lines of evidence suggest that PMN death is regulated by both intracellular death/survival signaling pathways and a variety of extracellular stimuli^3^. The general consensus is that many factors can delay constitutive neutrophil apoptosis, but nothing can completely prevent it^7^.

A growing body of evidence suggests that rather than being “transcriptional noise” and by functioning as primary regulators to control the expression of many target genes, numerous non-coding RNAs (ncRNAs) are involved in almost every aspect of cellular processes including differentiation, transcription, metabolism, and apoptosis^8,9^. As machine learning technology entered the bioinformatics field, computational models for non-coding RNAs research such as LRSSLMDA, IMCMDA, LRLSLDA and MDHGI^10–13^ have advanced greatly in recent years. Recent studies suggest that microRNAs (miRNAs) and long non-coding RNAs (lncRNAs) can regulate the lifespan of the short-lived myeloid cell^14,15^. For example, by regulating the transcription of pro-apoptotic gene Bcl2l11(Bim), the lncRNA Morrbid serves as a regulator of the lifespan of PMN^16^. It has also been reported that some lncRNA-related features could improve the diagnosis of some diseases such as non-small cell lung cancer, multiple myeloma, and bladder cancer^17–19^. It is therefore important to understand the regulatory role of lncRNAs in PMN apoptosis.

The timing of gene expression is important for apoptosis and therefore precisely controlled^20^. We first used different bioinformatics methods including short time-series expression miner (STEM) analysis screened statistically significant time-dependent gene expression profiles^21^. Then, using a time-series PMN apoptosis microarray datasets, we performed analyses of STEM and the lncRNA-mRNA co-expression network to identify key genes involved in PMN apoptosis. And finally, we experimentally confirmed the expression profiles of two mRNA (NFκB1 and BIRC3) and two lncRNAs (THAP9-AS1 andAL021707.6) during PMN spontaneous apoptosis in a time-dependent manner.

## Results

### Human spontaneous neutrophil apoptosis pattern

**Figure 1:**
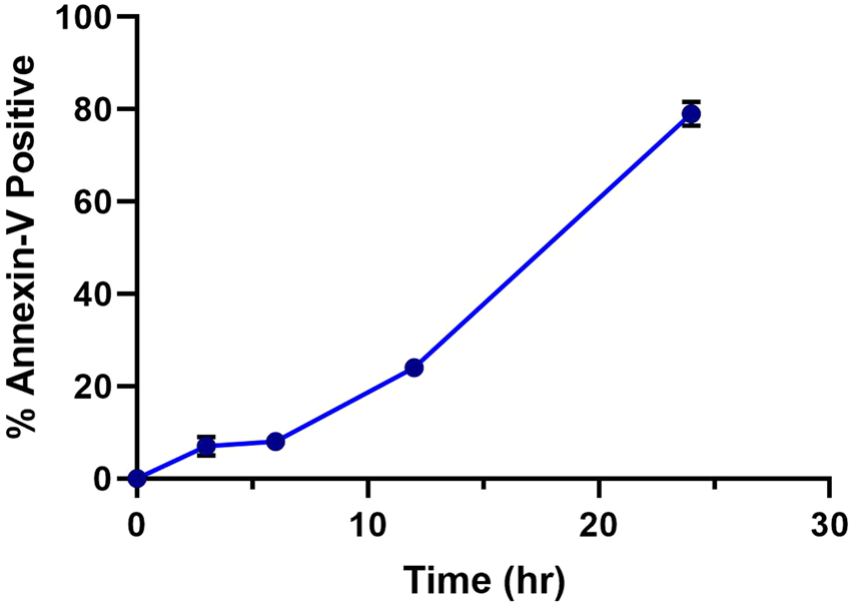
Human spontaneous neutrophil apoptosis. Apoptosis was assessed at the indicated time points (0, 3, 6, 12, 24 h) using Annexin V-FITC staining and flow cytometry. Pooled data are the mean ± SD (n = 4).

To obtain the spontaneous neutrophil apoptosis pattern, we performed flow cytometry to quantify apoptotic cells by measuring PS externalization on the PMN surface. Over time, PMN apoptosis increased gradually, having a relatively slow raise between 3 and 6 h, and a relatively quick increase after 6 h (Fig. 1).

### The baseline of microarray data and lncRNA expression profiles

**Figure 2:**
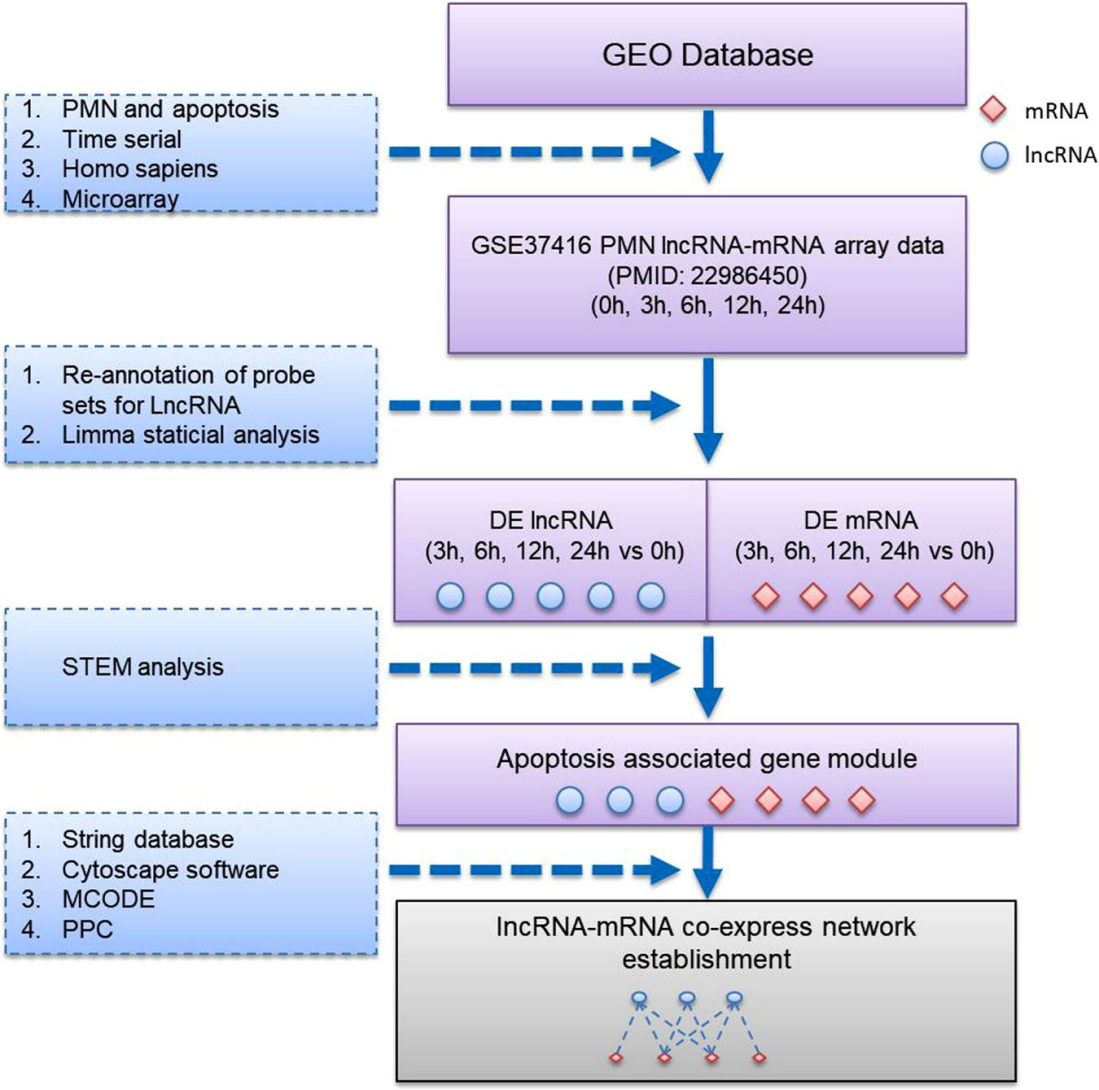
Data analysis flow diagram.

The public dataset GSE37416 was obtained from GEO and used in this study^22^. Twenty samples were selected from GSE37416 (GSM918524-GSM918527, GSM918532-GSM918535, GSM918540-GSM918543, GSM918548-GSM918551, and GSM918556-GSM918559), including samples at 0, 3, 6, 12, and 24 h (n = 4 each). The data analysis pipeline is shown in Fig. 2. A total of 4655 lncRNAs probe sets (matched with 4156 lncRNAs) were selected and re-annotated using the Affymetrix HG-U133 Plus 2.0 array from the BioMart database (Supplementary Table 1).

### Transcriptome changes of differentially expressed lncRNA and mRNA

**Figure 3:**
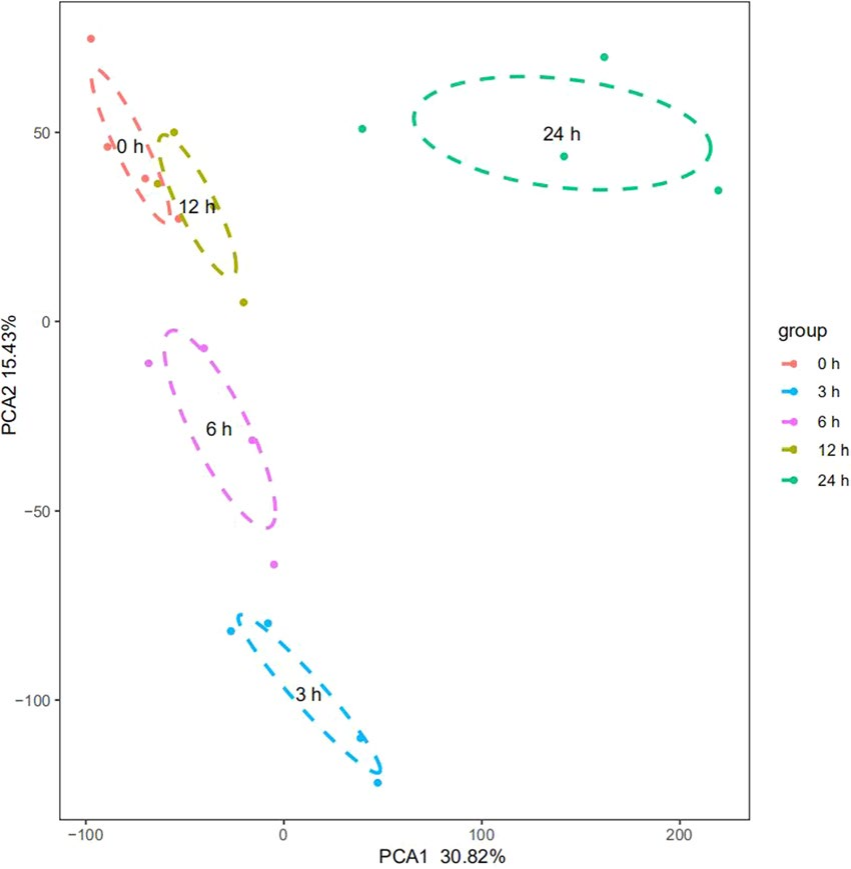
Summary of microarray data during apoptosis. PCA graph depicting variation and clusters of all genes in the data set at five time points in 2 dimensions.

**Figure 4:**
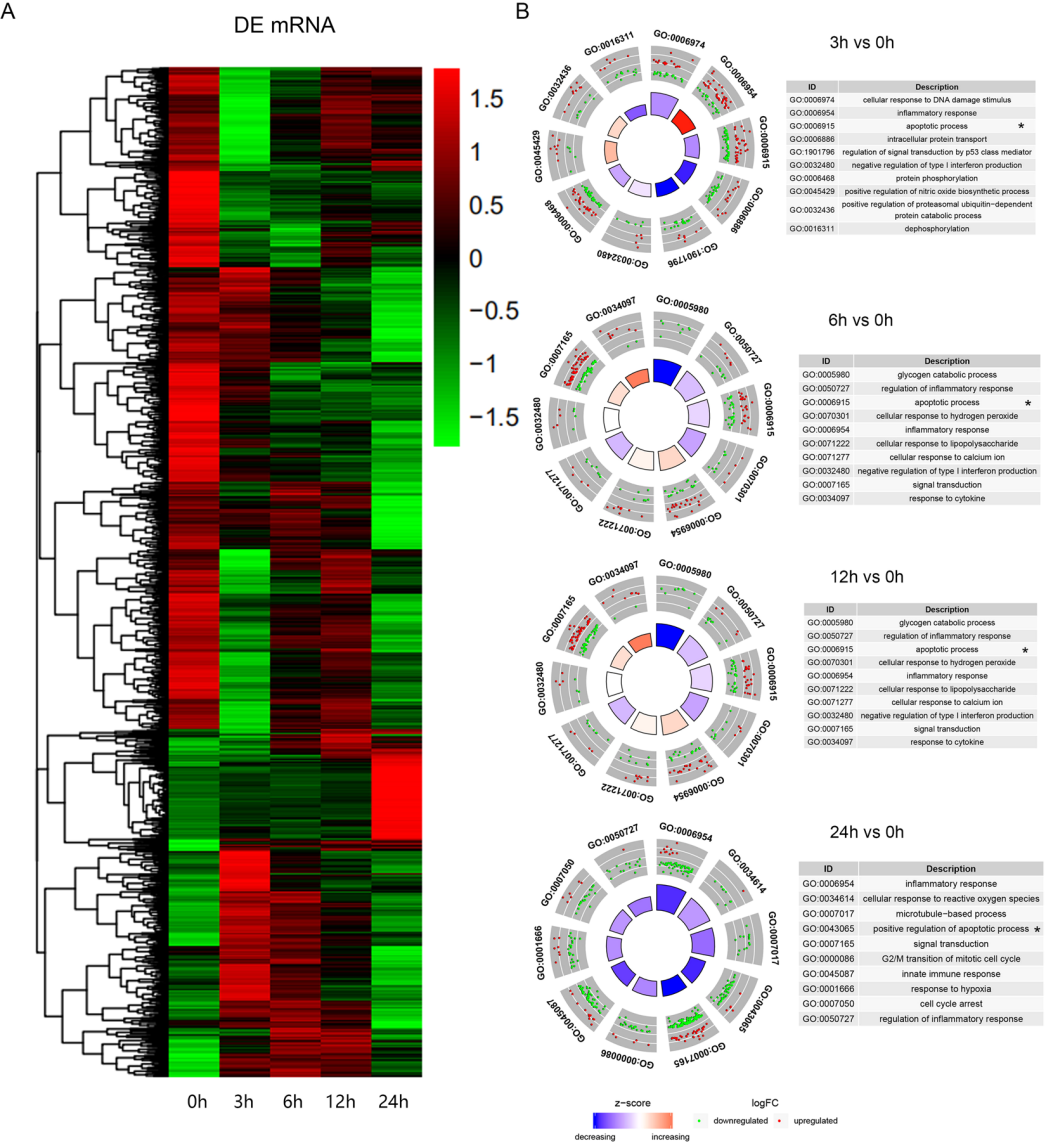
Overview of time-series mRNA differential expression (DE) analysis during neutrophil apoptosis. (**A**) Heatmap of differentially expressed genes for mRNAs. (**B**) Top GO terms significantly enriched in genes that are differentially expressed compared to 0 h as control.

**Figure 5:**
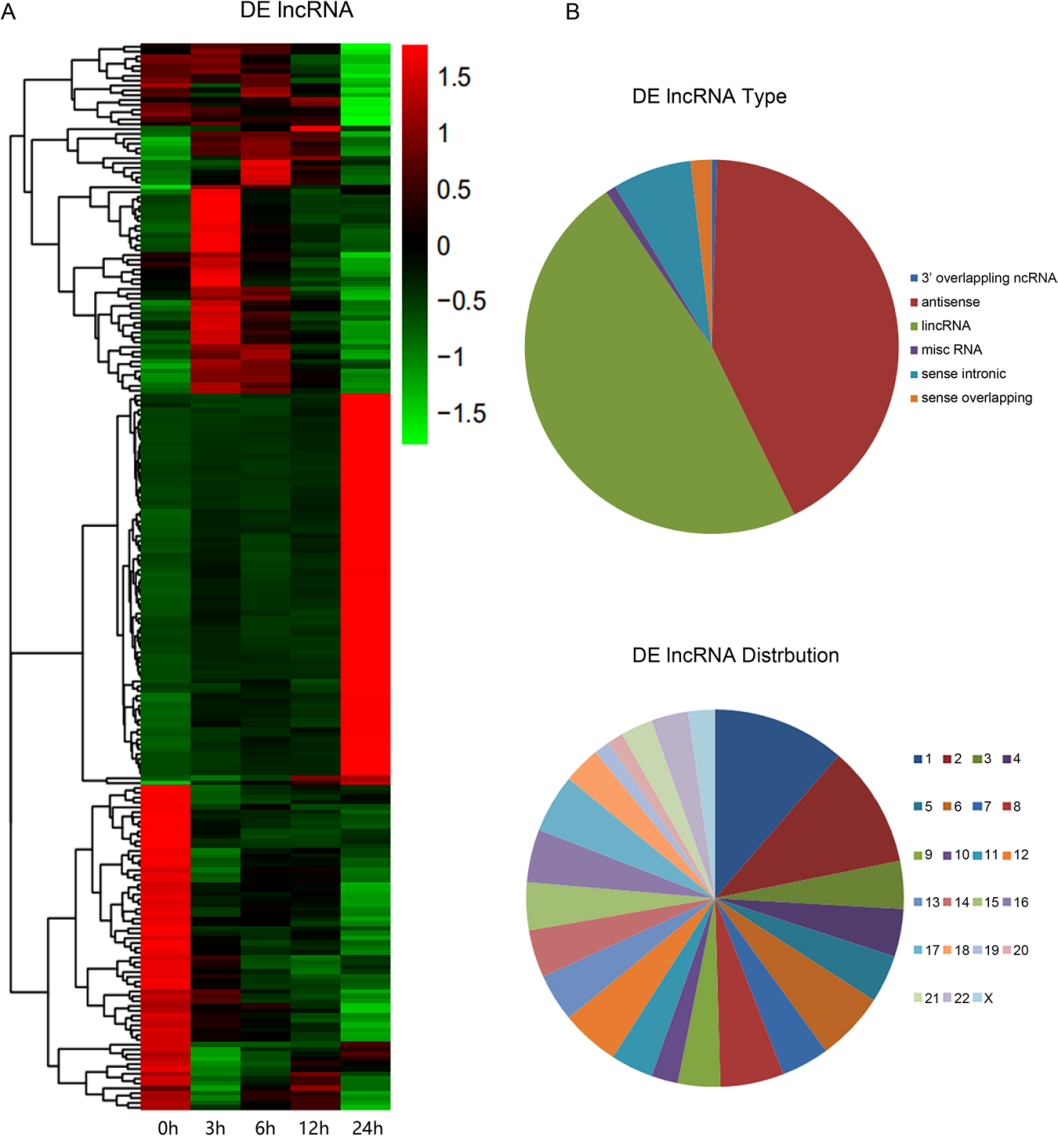
Overview of time-series lncRNA differential expression (DE) analysis during neutrophil apoptosis. (**A**) Heatmaps of differentially expressed genes for lncRNAs. (**B**) The transcript types and distribution of 220 differentially expressed lncRNAs.

PCA mapping was used to mathematically reduce the dimensionality of the spectrum of gene expression values and obtain information regarding the total amount of dataset variation^23^. The PCA data (Fig. 3) shows that the samples from 0, 3, 6, 12, and 24 h are clearly segregated from each other as apoptosis progressed. Microarray hybridization results showed that about 3050 mRNA and 220 lncRNA were differentially expressed when they were compared with 0 h as control (P < 0.01, logFC > 1). The differentially expressed mRNAs and Gene Ontology (GO) analyses at each time point are shown in Table 1 and Fig. 4, and the differentially expressed lncRNAs and transcript types and distribution were shown in Fig. 5. The majority of differentially expressed lncRNAs were antisense and long intergenic non-coding RNAs. These differentially expressed mRNAs were enriched mainly in the processes related to cellular response to DNA damage stimulus, apoptosis process, the inflammatory response, and catabolic processes.

**Table 1:** Overview of the differential expression analysis of time-series lncRNAs and mRNAs at the gene level.

Time point (h)	Up-regulation (n)	Down-regulation (n)	Total
**Differentially expressed lncRNAs (P** < **0.01, logFC** > **1)**			
3	41	30	71
6	29	26	55
12	12	25	37
24	81	80	161
Total (Unique)	163 (130)	161 (96)	324(220)
**Differentially expressed mRNAs (P** < **0.01, logFC** > **1)**			
3	577	1144	1721
6	416	631	1047
12	313	479	792
24	363	1349	1712
Total (unique)	1669 (1026)	3603 (2077)	5272 (3050)

### Temporal cluster analysis of significantly differentially expressed genes

**Figure 6:**
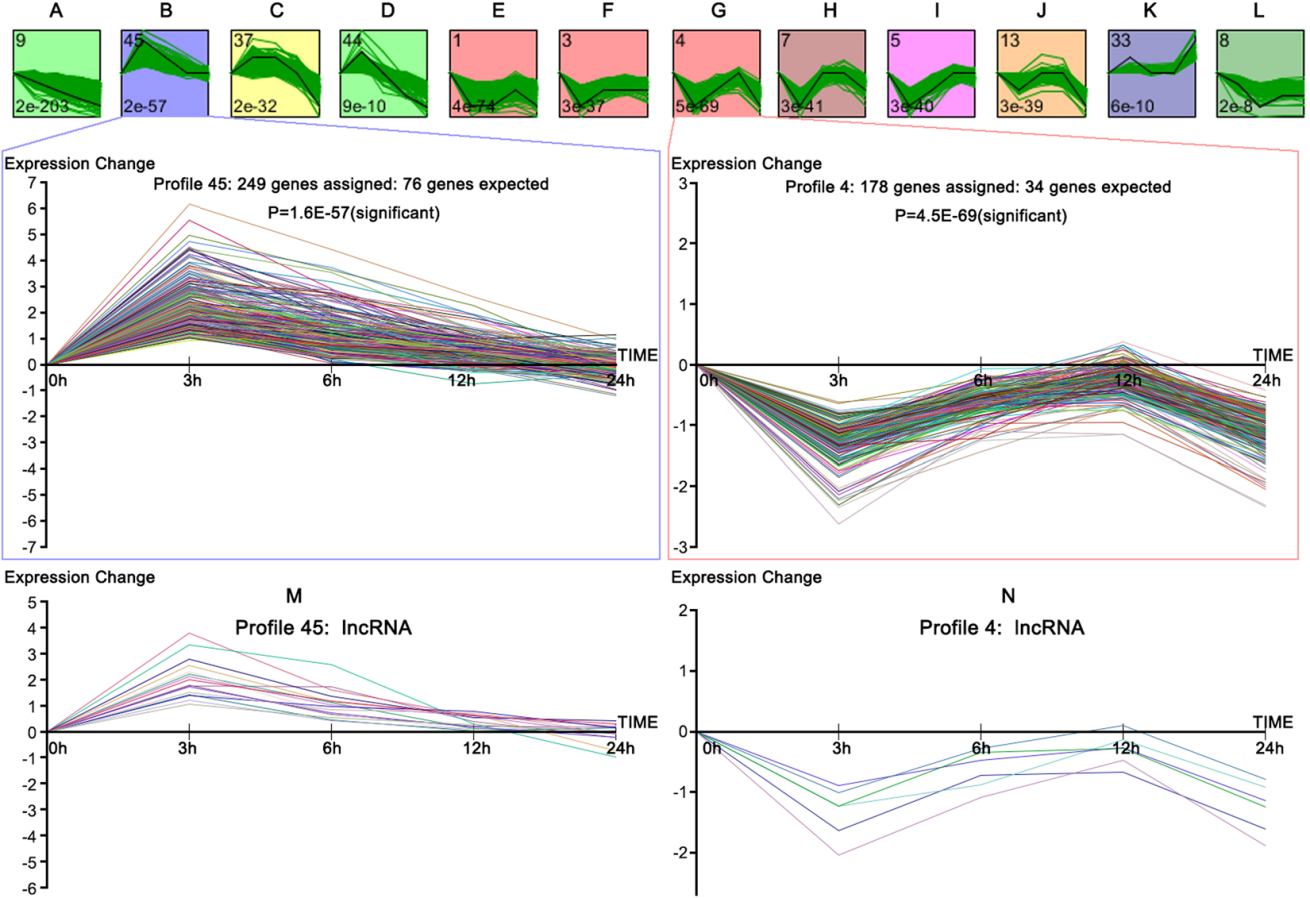
STEM clustering of the differentially expressed genes. (**A–L**) All significant profiles based on the P values of the numbers of genes assigned vs. expected. Profile 45 (0, 2, 1, 0, 0): 249 genes assigned, 76 genes expected, P-value = 1.6 E-57 (significant); Profile 4 (0, −2, −1, 0, −2): 178 genes assigned, 34 genes expected, P-value = 4.5E-69 (significant); (**M**) expression pattern of 16 lncRNAs assigned in Profile 45; **(N**) expression pattern of 6 lncRNAs assigned in Profile 4.

We normalized the expression data to 0 h (control) and identified temporal gene expression profiles using STEM. Within the 50 model profiles, we identified 12 significant clusters containing a total of 2342 genes (Fig. 6A–L). The profile boxes depict the gene expression patterns over five time points. The profile number on the top left corner of each profile box was assigned by STEM, and the number on the bottom left represents the adjusted P-value. We found genes with continuous downregulation pattern are in profile 9. Their expression increased in the early stage and dropped later, and they were distributed across profiles 45, 37, and 44. Meanwhile, profiles 1, 3, 4, 5, 7, 13, 33, and 8 showed biphasic expression patterns. GO function analysis for profiles shows that there are two interesting clusters comprised of genes related to apoptosis, namely profiles 4 and 45. The lncRNAs in the two profiles are depicted in Fig. 6M,N. Genes in profiles 4 and 45 are summarized in Supplementary Table 2. Additionally, the expression patterns of profiles 4 and 45 appear to be opposite to each other. GO analysis was performed for the genes in these two clusters based on the DAVID database. As shown in Table 2, the high-enrichment GO terms included: inflammatory response, NIK/NF-κB signaling, negative regulation of apoptotic signaling pathway, microtubule polymerization or depolymerization, execution phase of apoptosis, apoptotic mitochondrial changes, etc.

**Table 2:** GO results for profiles 4 and 45.

Profile	GO Function Category	Gene number	P value
45	inflammatory response	21	3.92E-08
	positive regulation of NF-kappaB transcription factor activity	12	6.73E-07
	I-kappaB kinase/NF-kappaB signaling	8	7.89E-06
	regulation of tumor necrosis factor-mediated signaling pathway	6	2.80E-05
	positive regulation of I-kappaB kinase/NF-kappaB signaling	10	1.57E-04
	negative regulation of apoptotic process	16	4.60E-04
	NIK/NF-kappaB signaling	6	0.001234782
4	microtubule polymerization or depolymerization	6	6.40E-04
	execution phase of apoptosis	5	1.10E-03
	response to osmotic stress	5	5.60E-03
	positive regulation of cell cycle arrest	5	6.90E-03
	apoptotic mitochondrial changes	5	0.01

### PPI network and lncRNA-mRNA co-expression network analyses for genes in profile 45 and 4

**Figure 7:**
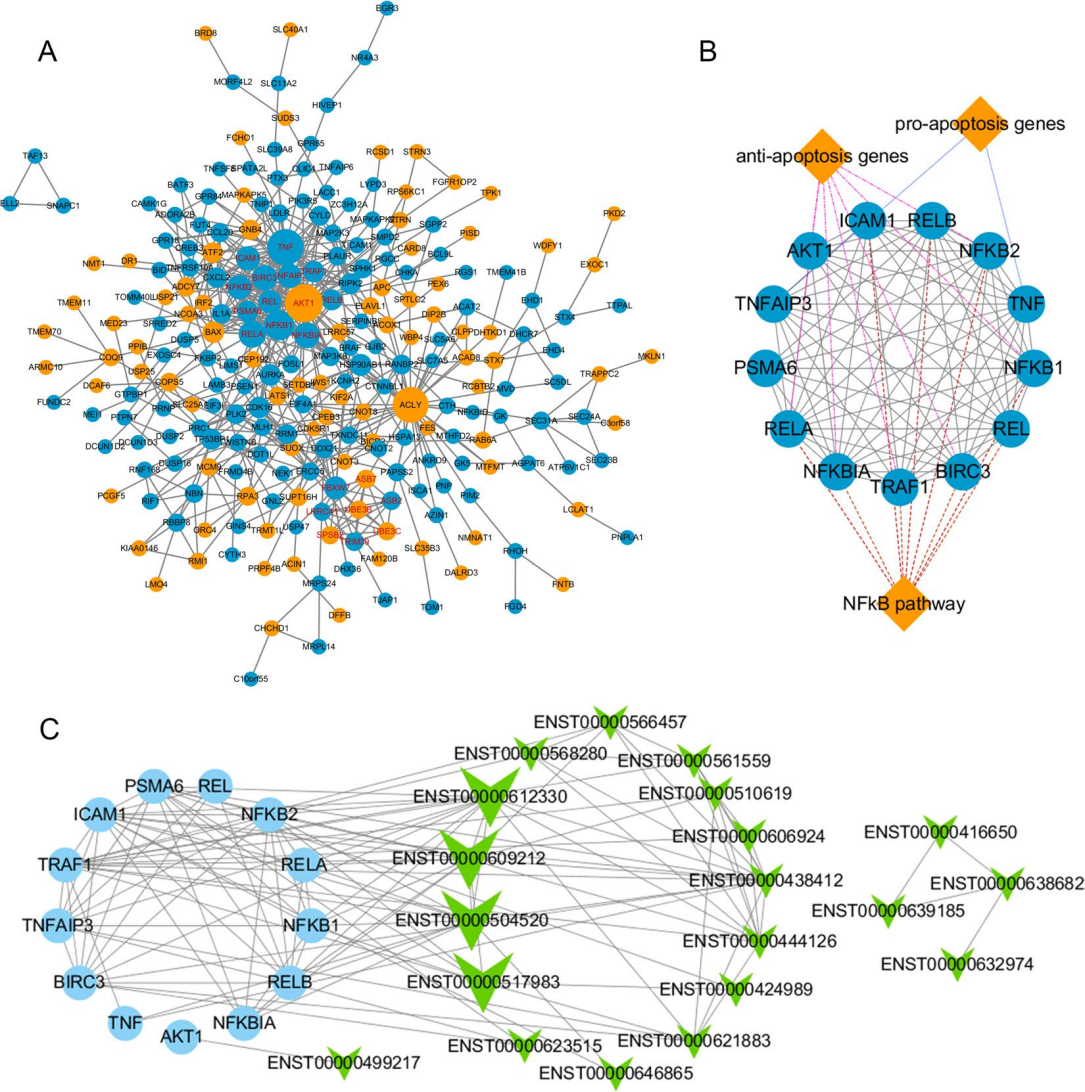
PPI network construction, module analysis, and lncRNA-mRNA co-expression network analyses. (**A**) The PPI network of genes in profiles 45 (light blue) and 4 (orange) was constructed using Cytoscape. (**B**) The hub gene module was obtained from the PPI network having 13 nodes and 71 edges with gene groups attached (diamonds). (**C**) The co-expression network was constructed with 13 hub mRNAs (light blue dots) and 20 co-expression lncRNAs (green arrowheads) in profiles 45 and 4.

The PPI network of differentially expressed genes was constructed by STRING and visualized by Cytoscape (Fig. 7A), and the most significant module was identified using Cytoscape plug-in MCODE (Fig. 7B). A lncRNA-mRNA co-expression network (Fig. 7C) was built to identify interactions between mRNAs and lncRNAs in profiles 4 and 45. The results showed that gene in these profiles highly interacted to each other, and hub genes in this module were mainly belonged to the NFκB signaling pathway such as NFKB1, RELA, BIRC2, and BIRC3. Among the co-expressed lncRNAs, we found two top-degree lncRNAs, ENST00000504520 (THAP9-AS1) and ENST00000609212 (AL021707.6), that had the highest Pearson’s correlation coefficients (>0.95) vs. NFκB1.

### Validation of the key spontaneous neutrophil apoptosis-associated mRNAs and lncRNAs

**Figure 8:**
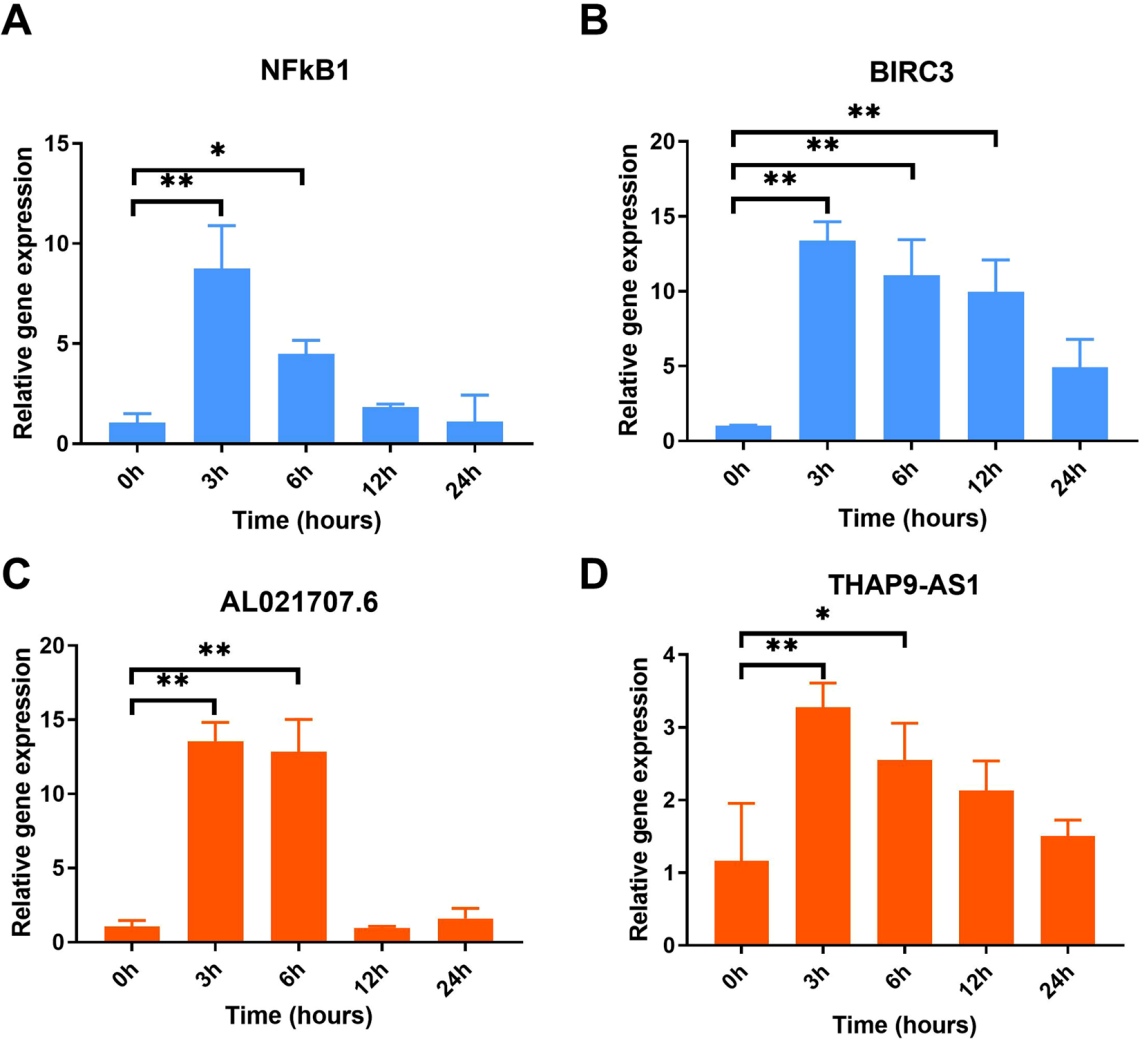
Validation of the expression of key genes. Neutrophils were purified and cultured for 0 h, 3 h, 6 h, 12 h and 24 h. qRT-PCR was performed to measure the relative expression of NFκB1 (**A**) and BIRC3 (**B**), and lncRNAs including AL021707.6 (**C**) and THAP9-AS1(**D**). β-ACTIN was used as an internal control. Experiments were performed three times. The t-test was used for analysis of each data set and comparison between different groups. *P* < 0.05 was considered as the level of statistical significance. *P < 0.05; **P < 0.01.

Finally, we wanted to experimentally validate the changes of bioinformatics-identified key mRNAs and lncRNAs during neutrophil spontaneous apoptosis. The qRT-PCR results (Fig. 8A,B) showed the levels of NFκB1 and BIRC3 increased dramatically during the first 3 hours of incubation and gradually decreased thereafter. Of note, these results are consistent with that from the microarray. Similarly, we estimated the changes of the two lncRNAs, THAP9-AS1 and AL021707.6, and found that these lncRNAs exhibited the exactly same pattern as NFκB1 and BIRC3 mRNAs (Fig. 8C,D).

## Discussion

Spontaneous neutrophil apoptosis plays critical roles in neutrophil homeostasis and resolving inflammation. Recently, many efforts have been made to decipher the molecular mechanisms of spontaneous PMN apoptosis. Most of the earlier studies have been only focusing on the roles of protein factors. More recent researches indicate that due to their extended lengths, lncRNAs could regulate protein expression by binding and sequestering their mRNAs. That is why some lncRNAs are also known as miRNA sponges^24^. It has been suggested that both miRNAs and lncRNAs are involved in the regulation of myeloid cell lifespan^14,15^. More recent findings suggest that lncRNA Morrbid is capable of affecting the lifespan of PMN by regulating the transcription of its neighboring pro-apoptotic gene, Bcl2l11 (Bim)^16^. Since gene expression dynamics are characterized by a time-dependent pattern, the expression profiles of genes at a single time point are insufficient to fully characterize the role of lncRNAs in apoptosis. We aimed to identify molecular events governing apoptosis using STEM to assess lncRNA and mRNA expression profiles.

Comparison of the mRNA transcriptional data of PMNs at 0 h and later time points, along with GO analysis, revealed differential expression of apoptosis-related genes. We also used the STEM platform to investigate how gene expression profiles change continuously during apoptosis process. We selected 50 predetermined temporal model profiles and quantified the genes assigned to each profile. Some distinct gene expression patterns were noticed during apoptosis. For example, genes modulating cell survival such as NFκB1 and BIRC3 in profile #45 significantly increased from hour 0 to hour 3 and reduced afterward. Meanwhile, genes involved in apoptosis in profile #4 significantly decreased between hour 0 and hour 3 and increased afterward. These findings are in consistent with that reported by Ward *et al*. that NFκB plays a central role in promoting PMN survival^25^. BIRC3, a member of the inhibitor of apoptosis (IAP) family, is at the downstream of NFκB signaling pathway and changed parallel to that of NFκB. BIRC3 is of the major anti-apoptosis genes and are partly responsible for sustained neutrophilia^26^. Mechanistically, IAP members can repress apoptosis by inhibiting caspase-3, -7, and -9^27,28^.

Through RT-PCR, we confirmed that at the early stage of apoptosis, NFκB1 is significantly upregulated along with its downstream pro-survival genes (especially BIRC3) and then gradually downregulated as apoptosis proceeds. And this regulation pattern may significantly affect the PMN apoptosis process, because the Annexin V-FITC and flow cytometry of PMN (Fig. 1) showed that PMN apoptosis rate decreased dramatically after the expression of NFκB1 and BIRC3 peaked at 3 h, and increased again when NFκB1 and BIRC3 reduced after 6 h. PPI network analysis of genes in profiles #45 and #4 also confirmed that genes in NFκB pathway are the hub of the apoptotic gene cluster. Together, these results indicate that PMN apoptosis may be strictly regulated by the levels of NFκB and IAP members. Moreover, using STEM algorithm and coexpression analysis, we identified two previously unreported lncRNAs that might be involved in the relation of apoptosis. THAP9-AS1 and AL021707.6 showed similar patterns as NFκB1 and IAP members with an extremely high correlation (PCC > 0.95). We hypothesize that these lncRNAs could be involved in regulating spontaneous neutrophil apoptosis by increasing the level of transcription factor NFκB, and its downstream IAP members to repress the caspases in the early stage of PMN spontaneous apoptosis (0–6 h) and when these lncRNAs were down-regulated at 12 h and 24 h the level of NFκB were also decreased sharply and PMN apoptosis occurs.

In summary, we presented a dynamic picture of the mRNAs and lncRNAs changes during neutrophil spontaneous apoptosis. Our study also has several limitations. For example, our findings are largely based on bioinformatics analysis of the publically available databases and therefore more functional experiments are needed to verify these results. The molecular mechanisms about how the NFkB-related genes affect neutrophil spontaneous apoptosis should also be further elucidated experimentally.

## Conclusions

We systematically analyzed mRNA and lncRNA expression changes in the spontaneous neutrophil apoptosis model. The results showed that the expression of both mRNAs and lncRNAs are stage-specific. NFκB1, BIRC3, and two co-expressed lncRNAs are inversely correlate with apoptosis. These findings suggest that these factor could be essential in the regulation of spontaneous neutrophil apoptosis. Finally, our finding not only bettered our understanding in PMN apoptosis but provided potential key regulatory molecule for PMN apotosis and therapeutic targets for over-reactive inflammatory response caused by the abnormality in PMN apoptosis.

## Methods

### Peripheral blood neutrophil isolation

Heparinized venous blood was obtained from three healthy individuals with the protocol approved by the Institutional Review Board for Human Subjects at Southwest Medical University. Written informed consent was obtained from every subject. All experiments were performed in accordance with relevant guidelines and regulations. Neutrophils were isolated using 3.0% Dextran T-500 (Amersham Biosciences Corp, Piscataway, NJ, USA), followed by density gradient centrifuge separation^29^. Neutrophils were suspended in phosphate-buffered saline, counted, and diluted to 1 × 10^7^/ml. Trypan blue, Wright-Giemsa staining, and microscopic analysis were performed to estimate neutrophil purity, and the suspensions were routinely >98% neutrophils. PMNs were diluted to 1 × 10^6^/ml in RPMI-1640 medium (10% fetal bovine serum; Gibco, Gaithersburg, MD, USA), transferred into T-25 flask and subsequently incubated at 37 °C with 5% CO_2_ for 12 h or 24 h.

### Detection of spontaneous neutrophil apoptosis using Annexin V *in vitro*

Spontaneous neutrophil apoptosis was assessed by measuring phosphatidylserine (PS) externalization on the neutrophil surface using Annexin V^30^. In brief, PMNs (5 × 10^6^/ml) were left untreated in RPMI-1640 medium. Aliquots of PMNs were removed at the indicated time points. An annexin V-fluorescein isothiocyanate (FITC)/propidium iodide (PI) apoptosis detection kit (KeyGen BioTech, Jiangsu, China) was used according to the manufacturer’s instructions.

### The baseline of microarray data

The Gene Expression Omnibus (GEO) database was searched to identify available datasets. After careful validation, the dataset GSE37416 containing neutrophil cell culture sampled at 0, 3, 6, 12, and 24 h were selected for this study^22^. The downloaded raw data were normalized using the log scale robust multi-array analysis with default settings^31^. All sample datasets were hybridized with the HG-U133 plus 2.0 Array (Affymetrix, Santa Clara, CA, USA), including 54,675 probe sets that are widely used in biological research (http://www.affymetrix.com/analysis/index.affx). Principal component analysis (PCA) was performed to visualize data variance^32^.

### lncRNA annotation pipeline

To obtain lncRNA expression data from the HG-U133 Plus 2.0 Array, the annotation was downloaded from the BioMart database (http://asia.ensembl.org/biomart/martview/). Only the probes annotated as lncRNAs were selected; transcript ID, chromosome location, strand, biologic types, and other annotation information were also downloaded^33^.

### Identification of differentially expressed lncRNA and mRNAs

The expression data of raw CEL files were normalized, log2 transformed and the background was adjusted utilizing a Bioconductor package Robust MultiArray Average (RMA)^34^, R 3.5.1 software. Next, a set of probe ID-centric gene expression values was retrieved for downstream analyses. The normalized data were then analyzed with linear models for microarray data (LIMMA), a modified *t*-test incorporating the Benjamini–Hochberg multiple hypotheses correction technique^35^. Probe sets without corresponding gene symbols or genes with more than one probe set were removed or collapsed to max probe through GSEA 3.0^36^. LogFC (fold change) >1 and adjusted P-values < 0.01 were considered statistically significant. A Gene Ontology (GO) analysis was performed using the Database for Annotation Visualization and Integrated Discovery (DAVID)^37^.

### STEM and GO analyses

We used the STEM^21^ clustering algorithm to identify temporal gene expression profiles during spontaneous neutrophil apoptosis with a maximum number of model profiles set as 50 and a maximum unit change in model profiles between time points set at 2. Gene expression values were transformed to log ratios relative to the expression value at 0 h. Then, each gene was assigned to the filtering criteria of the model profiles, and the correlation coefficient was determined. Standard hypothesis was performed using the true ordering of time points, and determined the p-value using the number of genes assigned to the model profile and the expected number of assigned genes (adjusted p-value, 0.05 by Bonferroni correction). The boxes in the figures were colored if the profiles were statistically significant. We also used DAVID v6.8 to identify the GO biological processes involving genes with significant profiles.

### PPI network construction, module analysis, and lncRNA-mRNA co-expression network analyses

The PPI network was constructed using the Search Tool for the Retrieval of Interacting Genes (STRING; http://string-db.org) (version 10.5) online database^38^. Interactions between proteins may offer insights into pathogenic mechanisms. In this study, the PPI network of DEGs from profiles 45 and 4 was constructed using the STRING database, and interaction with a combined score >0.4 was considered statistically significant. Cytoscape (version 3.6.1) is an open source bioinformatics software platform for visualizing molecular interaction networks^39^. The Cytoscape plug-in Molecular Complex Detection (MCODE) (version 1.5.1) is an algorithm for clustering a given network based on the topology to identify densely connected regions^40^. The PPI networks were constructed using Cytoscape, and the most significant module in the PPI networks was identified using MCODE. The selection criteria were as follows: degree cut-off = 2, MCODE scores >7, Max depth = 100, node score cut-off = 0.2, and k-score = 2. It is believed that genes with the same biological function or that regulate the same pathway may have similar expression patterns. The lncRNA-mRNA co-expression network was built based on the Pearson correlation coefficient (PCC) analysis of the lncRNA and mRNA expression levels^41^. The PCC was calculated for each lncRNA-mRNA, mRNA-mRNA, and lncRNA-lncRNA pair using package diffcoexp in R software^42^, and significant lncRNA-mRNA pairs with the absolute PCC > 0.9 and q-value < 0.01 were selected to construct the co-expression network using the Cytoscape 3.6.1 program.

### Quantitative real-time PCR

Total RNA was extracted using TRIzol reagent followed by reverse transcription and quantitative real-time polymerase chain reaction (qRT-PCR) using the Power SYBR Green PCR Mastermix (Applied Biosystems, Foster City, CA, USA) as described previously^43^. The mean expression values were calculated based on the mean expression of the housekeeping gene β-actin. The primers used in this research are listed in Table 3. Statistical analyses were done using one-way analysis of variance, followed by Student–Newman–Keuls posthoc tests using SPSS 16.0 software (SPSS Inc., Chicago, IL, USA).

**Table 3:** The qRT-PCR primers used in this research.

	Forward	Reverse
β-ACTIN	5′-CGACAGTCAGCCGCATCTT-3′	5′-CCAATACGACCAAATCCGTTG-3′
NFΚB1	5′-CAAGGACATGGTGGTCGGCTTC-3′	5′-CGCCTCTGTCATTCGTGCTTCC-3′
BIRC3	5′-TCTCTGCAAGAAGCTGAAGCTGTG-3	5′-TCTCCGCAATTGTTCTTCCACTGG-3′
THAP9-AS1	5′-ATACGCACTGGTGGAAGGAGAGG-3′	5′-AAGTGGTCGTGATTCATGCTGTCG-3′
AL021707.6	5′-CCAGAGACGTTCCAGCATAAGGC-3′	5′-AGACTTCGGTACTGTGTCCTCAGG-3′
CXCL8	5′-GAGAGTGATTGAGAGTGGACCAC-3′	5′-CACAACCCTCTGCACCCAGTTT-3′

## Supplementary information

Supplementary Dataset 1

Supplementary Dataset 2

## References

[1] Luo HR, Loison F (2008). Constitutive neutrophil apoptosis: mechanisms and regulation. American journal of hematology.

[2] Scheel-Toellner D (2004). Early events in spontaneous neutrophil apoptosis. Biochemical Society transactions.

[3] Geering B, Simon HU (2011). Peculiarities of cell death mechanisms in neutrophils. Cell Death Differ.

[4] Simon HU (2003). Neutrophil apoptosis pathways and their modifications in inflammation. Immunol Rev.

[5] Jack RM, Fearon DT (1988). Selective synthesis of mRNA and proteins by human peripheral blood neutrophils. J Immunol.

[6] Kobayashi SD, DeLeo FR (2009). Role of neutrophils in innate immunity: a systems biology-level approach. Wiley Interdiscip Rev Syst Biol Med.

[7] Witko-Sarsat V, Pederzoli-Ribeil M, Hirsch E, Sozzani S, Cassatella MA (2011). Regulating neutrophil apoptosis: new players enter the game. Trends Immunol.

[8] Chen X, Yan CC, Zhang X, You ZH (2017). Long non-coding RNAs and complex diseases: from experimental results to computational models. Brief Bioinform.

[9] Chen X, Xie D, Zhao Q, You ZH (2019). MicroRNAs and complex diseases: from experimental results to computational models. Brief Bioinform.

[10] Chen X, Yin J, Qu J, Huang L (2018). MDHGI: Matrix Decomposition and Heterogeneous Graph Inference for miRNA-disease association prediction. PLoS Comput Biol.

[11] Chen X, Wang L, Qu J, Guan NN, Li JQ (2018). Predicting miRNA-disease association based on inductive matrix completion. Bioinformatics (Oxford, England).

[12] Chen X, Yan GY (2013). Novel human lncRNA-disease association inference based on lncRNA expression profiles. Bioinformatics (Oxford, England).

[13] Chen X, Huang L (2017). LRSSLMDA: Laplacian Regularized Sparse Subspace Learning for MiRNA-Disease Association prediction. PLoS Comput Biol.

[14] Hutcheson R (2015). miR-21-mediated decreased neutrophil apoptosis is a determinant of impaired coronary collateral growth in metabolic syndrome. American journal of physiology. Heart and circulatory physiology.

[15] Zhao H, Zhang X, Frazao JB, Condino-Neto A, Newburger PE (2013). HOX antisense lincRNA HOXA-AS2 is an apoptosis repressor in all trans retinoic acid treated NB4 promyelocytic leukemia cells. J Cell Biochem.

[16] Kotzin JJ (2016). The long non-coding RNA Morrbid regulates Bim and short-lived myeloid cell lifespan. Nature.

[17] Shen X (2017). Upregulated lncRNA-PCAT1 is closely related to clinical diagnosis of multiple myeloma as a predictive biomarker in serum. Cancer Biomark.

[18] Wang HM, Lu JH, Chen WY, Gu AQ (2015). Upregulated lncRNA-UCA1 contributes to progression of lung cancer and is closely related to clinical diagnosis as a predictive biomarker in plasma. Int J Clin Exp Med.

[19] Zhang Shujun, Du Lutao, Wang Lishui, Jiang Xiumei, Zhan Yao, Li Juan, Yan Keqiang, Duan Weili, Zhao Yinghui, Wang Lili, Wang Yunshan, Shi Yuliang, Wang Chuanxin (2018). Evaluation of serum exosomal LncRNA-based biomarker panel for diagnosis and recurrence prediction of bladder cancer. Journal of Cellular and Molecular Medicine.

[20] Gabelloni ML, Trevani AS, Sabatte J, Geffner J (2013). Mechanisms regulating neutrophil survival and cell death. Semin Immunopathol.

[21] Ernst J, Bar-Joseph Z (2006). STEM: a tool for the analysis of short time series gene expression data. BMC Bioinformatics.

[22] Schwartz JT (2013). Francisella tularensis alters human neutrophil gene expression: insights into the molecular basis of delayed neutrophil apoptosis. Journal of innate immunity.

[23] Hubert M, Engelen S, Robust PCA (2004). and classification in biosciences. Bioinformatics (Oxford, England).

[24] Wilkes MC, Repellin CE, Sakamoto KM (2017). Beyond mRNA: The role of non-coding RNAs in normal and aberrant hematopoiesis. Mol Genet Metab.

[25] Ward C (1999). NF-kappaB activation is a critical regulator of human granulocyte apoptosis *in vitro*. J Biol Chem.

[26] Hasegawa T (2003). Expression of the inhibitor of apoptosis (IAP) family members in human neutrophils: up-regulation of cIAP2 by granulocyte colony-stimulating factor and overexpression of cIAP2 in chronic neutrophilic leukemia. Blood.

[27] Dubrez-Daloz L, Dupoux A, Cartier J (2008). IAPs: more than just inhibitors of apoptosis proteins. Cell Cycle.

[28] Uren AG, Pakusch M, Hawkins CJ, Puls KL, Vaux DL (1996). Cloning and expression of apoptosis inhibitory protein homologs that function to inhibit apoptosis and/or bind tumor necrosis factor receptor-associated factors. Proc Natl Acad Sci USA.

[29] Nauseef WM (2007). Isolation of human neutrophils from venous blood. Methods Mol Biol.

[30] Schwartz JT (2012). Francisella tularensis inhibits the intrinsic and extrinsic pathways to delay constitutive apoptosis and prolong human neutrophil lifespan. J Immunol.

[31] Bolstad BM, Irizarry RA, Astrand M, Speed TP (2003). A comparison of normalization methods for high density oligonucleotide array data based on variance and bias. Bioinformatics (Oxford, England).

[32] Ligges, U. & Maechler, M. Scatterplot3d - An R Package for Visualizing Multivariate Data. *Journal of Statistical Software* **8**, http://hdl.handle.net/10 (2003).

[33] Zhang X (2012). Long non-coding RNA expression profiles predict clinical phenotypes in glioma. Neurobiol Dis.

[34] Irizarry RA (2003). Exploration, normalization, and summaries of high density oligonucleotide array probe level data. Biostatistics.

[35] Smyth GK (2004). Linear models and empirical bayes methods for assessing differential expression in microarray experiments. Stat Appl Genet Mol Biol.

[36] Subramanian A (2005). Gene set enrichment analysis: a knowledge-based approach for interpreting genome-wide expression profiles. Proc Natl Acad Sci USA.

[37] Huang DW (2007). The DAVID Gene Functional Classification Tool: a novel biological module-centric algorithm to functionally analyze large gene lists. Genome Biol.

[38] Franceschini A (2013). STRING v9.1: protein-protein interaction networks, with increased coverage and integration. Nucleic acids research.

[39] Smoot ME, Ono K, Ruscheinski J, Wang PL, Ideker T (2011). Cytoscape 2.8: new features for data integration and network visualization. Bioinformatics (Oxford, England).

[40] Bandettini WP (2012). MultiContrast Delayed Enhancement (MCODE) improves detection of subendocardial myocardial infarction by late gadolinium enhancement cardiovascular magnetic resonance: a clinical validation study. Journal of cardiovascular magnetic resonance: official journal of the Society for Cardiovascular Magnetic Resonance.

[41] Prieto C, Risueno A, Fontanillo C, De las Rivas J (2008). Human gene coexpression landscape: confident network derived from tissue transcriptomic profiles. PloS one.

[42] Wei, W. diffcoexp: Differential Co-expression Analysis (2018).

[43] Zheng Y, Jia L (2016). Long noncoding RNAs related to the odontogenic potential of dental mesenchymal cells in mice. Arch Oral Biol.

